# Supplementary material for: Identification of photoperiod-induced specific miRNAs in the adrenal glands of Sunite sheep (Ovis aries)
Source: Front Vet Sci. 2022 Jul 22;9:888207. doi: 10.3389/fvets.2022.888207 (PMC9354845; doi:10.3389/fvets.2022.888207)
Supplement: Supplementary file 1 [file Data_Sheet_1.ZIP › Supplementary file/miRDeep2 file.zip!/miRDeep2 file/Know_Structure/SP42A2/oar-mir-3955.pdf]

miRBase precursor : oar-mir-3955
Total read count : 632
oar-miR-3955-5p read count : 581
oar-miR-3955-3p read count : 35
remaining reads : 16

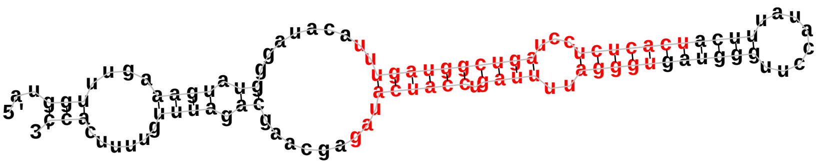

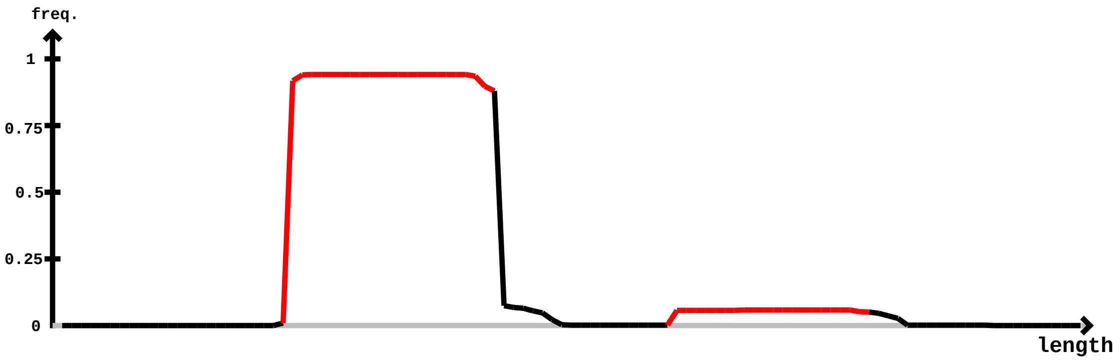

oar-miR-3955-5p

oar-miR-3955-3p

```
5'- augguuugaaaguaugggauacauuugauggcugauccucucacuacuuuauaccuuggguagugggauuuuaguccaucauagagcaagcagauuuguuuucacc  -3'   exp
    ...........((((...))))...((((((((((...(((((((((((........)))))))))))..)))).)))))).(((((((......)))))))....    reads   mm    sample
    ......................auuugauggcugauccucucacu.............................................................    6       0     seq
    .......................uuugauggcugauccucuc................................................................    4       0     seq
    .......................uuugauggcugauccucuca...............................................................    24      0     seq
    .......................uuugauggcugauccucucac..............................................................    11      0     seq
    .......................uuugauggcugUuccucucacu.............................................................    1       1     seq
    .......................uuugauggcugauccucucacu.............................................................    490     0     seq
    .......................uuugauggcugauccucucacua............................................................    4       0     seq
    .......................uuugauggcugauccucucacuac...........................................................    2       0     seq
    .......................uuugauggcugauccucucacuacu..........................................................    5       0     seq
    .......................uuugauggcugauccucucacuacuu.........................................................    5       0     seq
    .......................uuugauggcugauccucucacuacuuu........................................................    14      0     seq
    .......................uuugauggcugauccucucacuacuuua.......................................................    13      0     seq
    .......................uuugauggcugauccucucacuacuuuau......................................................    1       0     seq
    ........................uugauggcugauccucucacu.............................................................    12      0     seq
    ........................uugauggcugauccucucacuacu..........................................................    1       0     seq
    ........................uugauggcugauccucucacuacuuu........................................................    1       0     seq
    .........................ugauggcugauccucucacuacuuu........................................................    1       0     seq
    ...................................................uaccuuggguagugggauuuuaguccaucau........................    1       0     seq
    ...............................................................ugggauuuuaguccaucaC........................    1       1     seq
    ...............................................................ugggauuuuaguccaucau........................    2       0     seq
    ...............................................................ugggauuuuaguccaucaua.......................    1       0     seq
    ...............................................................ugggauuuuaguccaucauag......................    3       0     seq
    ...............................................................ugggauuuuaguccaucauaga.....................    6       0     seq
    ...............................................................ugggauuuuaguccaucauagag....................    6       0     seq
    ...............................................................ugggauuuuaguccaucauagagc...................    16      0     seq
    ......................................................................uuaguccaucauagagcaagcagauu..........    1       0     seq
```
